# The Management of Acne

**Published:** 1903-07-25

**Authors:** 


### THE MANAGEMENT OF ACNE.

A discussion recently took place among a number of skin specialists at the New York Post Graduate Clinical Society [1] on a subject with regard to which medical men are often consulted, namely the management of cases of acne. It is one of the commonest diseases, and though in many cases it is one of very minor importance, it is always a source of annoyance to the patient, and neglect or improper treatment may lead to more or less marked and permanent disfigurement.

The lesions consist of comedones, papules, and pustules, which commonly co-exist in all cases. The distribution is principally on the forehead, nose, cheeks, and chin, but the shoulders, back, and chest are often involved and frequently remain so when the face is free. The affection usually begins about puberty and has a natural tendency to diminish about the twenty-fifth year.

Predisposing causes are lowered vitality, with a sluggish circulation consequent on rapid growth and imperfect hygiene. It should be noted that about the age of puberty the glandular tissues are naturally very active while the subject enjoys more freedom from parental control, and is subjected to less restraint than previously as regards diet, hours, and habits.

Indigestion, together with bowel torpor, is nearly always present, though the former may be so slight as to have escaped the patient's notice. Careful inquiry, however, will lead to a history of more or less discomfort after meals, such as sleepiness, weight, and fulness, even though the more obvious discomfort of pain be absent. Sweetmeats, pastry, and pickles are commonly found to figure largely in the dietary. In young women menstrual irregularity is also a contributing cause, and the lesions are often most abundant at the monthly epochs.

The first step to take in the treatment of every case alike is to regulate the diet, and it generally saves time and trouble to obtain a complete list of everything in the way of food or drink which the patient is *likely* to take, and strike out from it everything which, under the circumstances, is considered objectionable. In a majority of cases, the use of tea, coffee, and alcoholic liquors, if not absolutely forbidden, should be very strictly limited. Pastry, pickles, sweetmeats and greasy foods should be prohibited entirely. The general hygiene of the conditions under which the patient lives should be inquired into, and adequate exercise in the open air insisted upon as well as free ventilation of sleeping apartments and working rooms. The personal "grooming" of the patient should be so thorough as to enable the skin of the whole body to perform its function without hindrance. Regular tepid soap baths are necessary for this purpose, but the cold douche followed by vigorous friction does so much to improve the general circulation that it is also to be advised. The majority of these patients also present a condition of a more or less coated tongue and other evidences of torpid liver and anæmia, and these must be met by suitable stomachic remedies, followed up by quinine, iron, and other tonics. In a certain number of cases of recent origin, this general treatment will alone suffice, but local treatment is what the patient expects, and is also what is commonly required, in addition to the systemic measures before recommended. Practically the first local indication is removal of the comedones, which may be effected in various ways. The better way of getting the skin free from them without the use of instruments, is vigorous pinching and kneading of the skin after friction with green soap dissolved in alcohol. This done, and repeated at intervals as often as required, a simple ointment of cold cream and borax should be gently rubbed in.

In the milder cases, where the principal lesions consist of multiple comedones and a few superficial pustules, no more local treatment than the foregoing is called for. In other cases, however, where the pustules are many and there are numerous deep-seated and indurated papules and some deep-seated abscesses, the treatment must be more active.

Curetting of the papules may be performed, but is rarely necessary and is, of course, strongly objected to by patients. A better method is by the application of a stimulating lotion of which the following is the formula :—

℞ zinci sulph. ... ℥i.

Potass. sulphuret. ℥i.

Sulph. præcipit. ℥i.

Alcohol... ... q.s.

Aq. rosæ ad ℥iv.

Under this application the skin peels with a rapidity which varies in different cases. In those in which the peeling appears to be progressing with undue rapidity the stimulation should be stopped for a day or two, and the cold cream and borax ointment substituted.

The deep-seated nodules require more energetic stimulation, and the following preparation is the best for this purpose :—

℞ glycerine.
Sulphur præcip.
Potass. Carb.
Equal parts of each, rubbed into a paste.

This is very powerful and must be applied over nodules only and nowhere else. Absorption in most cases follows its use, but if not, and pus forms or has formed before treatment is commenced, it must be let out with the point of a bistoury, and a tiny drop of pure carbolic introduced on a fine probe. The patient should be warned that a slight scar is inevitable.

[1] The Postgraduate Journal.

